# Venous thromboembolism in children with cancer – A population-based cohort study^☆^^☆☆^

**DOI:** 10.1016/j.thromres.2013.12.021

**Published:** 2014-03

**Authors:** Alex J. Walker, Matthew J. Grainge, Tim R. Card, Joe West, Susanna Ranta, Jonas F. Ludvigsson

**Affiliations:** a Division of Epidemiology and Public Health, University of Nottingham, Nottingham City Hospital, NG5 1PB UK; b Nottingham Digestive Diseases Centre, NIHR Biomedical Research Unit; c Childhood Cancer Research Unit, Karolinska Institutet Stockholm Sweden; d Department of Medical Epidemiology and Biostatistics, Karolinska Institutet Sockholm Sweden; e Department of Pediatrics, Örebro University Hospital, Örebro University, Örebro Sweden

**Keywords:** CI, Confidence Interval, CVC, Central venous catheters, HR, Hazard ratio, PE, Pulmonary embolism, VTE, Venous thromboembolism, Cancer, Malignancy, Thromboembolism, Venous thrombosis, Children

## Abstract

**Introduction:**

Cancer is a known risk factor for venous thromboembolism (VTE) in adults, but population-based data in children are scarce.

**Materials and methods:**

We conducted a cohort study utilising linkage of the Clinical Practice Research Database (primary care), Hospital Episodes Statistics (secondary care), UK Cancer Registry data and Office for National Statistics cause of death data. From these databases, we selected 498 children with cancer diagnosed between 1997 and 2006 and 20,810 controls without cancer. We calculated VTE incidence rates in children with cancer vs. controls, and hazard ratios (HRs) using Cox regression.

**Results:**

We identified four VTE events in children with cancer compared with four events in the larger control population corresponding to absolute risks of 1.52 and 0.06 per 1000 person-years respectively. The four children with VTE and cancer were diagnosed with hematological, bone or non-specified cancer. Childhood cancer was hence associated with a highly increased risk of VTE (HR adjusted for age and sex: 28.3; 95%CI = 7.0-114.5).

**Conclusions:**

Children with cancer are at increased relative risk of VTE compared to those without cancer. Physicians could consider thromboprophylaxis in children with cancer to reduce their excess risk of VTE however the absolute risk is extremely small and the benefit gained therefore would need to be balanced against the risk invoked of implementing such a strategy.

**Novelty & Impact Statements:**

While there is a reasonable level of knowledge about the risk of VTE in adult populations, it is not well known whether this risk is reflected in paediatric patients. We found a substantial increase in risk of VTE in children with cancer compared to a child population without cancer. While this finding is important, the absolute risk of VTE is still low and must be balanced with the risks of anticoagulation.

## Introduction

Cancer is a leading cause of death in children in the Western world. In the last 30 years, the survival rate has however improved dramatically, and today the 5-year-survival of both leukemia and Non Hodgkins Lymphoma in children exceeds 85% [1]. With increasing survival rates, health care in these children focuses more on the prevention of complications from cancer and cancer treatment. One such complication is venous thromboembolism (VTE) [2–7]. VTE, defined as deep-vein thrombosis or pulmonary embolism, is a leading cause of non-cancer death in adult patients with cancer [8].

As the treatment strategies for critically ill children have improved, the rate of VTE in children has increased both in the general population [9], and among patients with cancer [10]. VTE is also associated with a substantial excess mortality [9,10] and seems to influence cancer mortality even when tumour stage and cancer regimen have been taken into consideration [11]. In a recent US study [9], Boulet et al. reported that venous catheter use, mechanical ventilation, malignancy, and hospitalization for at least five days were all risk factors for VTE-related hospital admissions. Despite the identification of these risk factors, few studies have quantified the absolute and relative risks of VTE in cancer compared with general population controls. We recently showed that adults with cancer are at a 4-5-fold increased risk of VTE compared to the general population [12]. Guidelines for adults stipulate that thromboprophylaxis is advised for high-risk inpatients including those with cancer. Whilst routine prophylaxis is not advised for outpatients recent updates to U.S. guidelines advise that prophylaxis is recommended for patients with both cancer and additional risk factors for thrombosis providing they are at low risk of bleeding [13,14]. However it is not clear if children with malignancies might benefit from thromboprophylaxis [2].

The aim of the current study was to examine the risk of VTE in children with cancer, using population-based English data.

## Materials and Methods

We utilised population-based health registers to investigate the risk of VTE in cancer patients under the age of 18 years from England (such patients are hereby denoted “children”). Our cohort comprised children who had linked data available from all three data sources described below. A more detailed description of our methods, has been published elsewhere [12].

### Cancer Registry Data

Information on cancer diagnoses was obtained from the National Cancer Intelligence Network (NCIN), which processes data supplied by all regional cancer registries in the United Kingdom. Two related but separate databases make up the cancer registry data; the Merged Cancer Registry data (1990 to 2006, from English registries only) and the Office of National Statistics (ONS) minimum cancer dataset (1971 to 2006). From these sources, we selected children with cancer diagnosed between April 1997 and December 2006 as this was the period from which data linked to Clinical Practice Research Datalink (CPRD) and Hospital Episodes Statistics (HES) were available. Cancers were classified into 10 categories according to Cancer Research UK incidence data. Cancers diagnosed outside these categories were referred to an 11th non-specified cancer category (“other site”).

### Clinical Practice Research Datalink (CPRD)

Through the CPRD (formerly known as the General Practice Research Database, GPRD), we were able to ascertain data on VTE. The CPRD is an anonymised primary care database that was started in 1987 and now encompasses some 600 GP UK practices. This database contains all recorded primary care data including clinical diagnoses, treatments and outcomes. Data from the CPRD has been found to be broadly representative of the UK population with regards to sex, age, socio-economic status and geographic location [15], whilst the validity of coding has been demonstrated across a range of medical conditions [16].

### Hospital Episodes Statistics (HES)

The third database used in this paper is the Hospital Episodes Statistics (HES) database. This is a secondary care database that enlists all hospital admissions in England. For each inpatient episode we collected data on all diagnoses and procedures. About half of the CPRD practices are linked to the HES and cancer registry databases.

### Exclusion Criteria

We excluded patients who (I) were from a CPRD practice that was not linked to the HES and cancer registry databases; (II) had received their cancer diagnosis outside the HES and CPRD registration dates; (III) were diagnosed with cancer within one year of registration at a participating general practice; (IV) had a VTE diagnosis at any point prior to the date of first cancer diagnosis. Finally we excluded (V) all individuals with a non-melanoma skin cancer.

### Comparison Cohort

The general population comparison cohort was identified from the CPRD. In order to maximize statistical power, all available controls without a diagnosis of cancer were eligible. Controls then received a pseudo-diagnosis date generated at random within the registration period for each patient. Any control whose pseudo-diagnosis date was after they reached 18 years of age was then excluded.

### VTE

Our outcome, VTE; was defined according to relevant ICD codes (I26.0, I26.9, I80, I80.1-I80.9, I81, I82, I82.0-I82.9) in HES and Read codes mapped to these in the CPRD, if supported by any of the following: (I) a prescription for an anticoagulant or evidence of anticoagulation (based on Read codes) between 15 days before and 90 days after the VTE event, or (II) when the VTE was followed by death within 30 days of the VTE diagnosis. We also accepted VTE when listed as the underlying cause of death. Earlier data indicate that VTE defined according to primary care data has a high validity [17].

### Statistics

Follow-up started at cancer diagnosis in cases or at pseudo-diagnosis in controls respectively. It ended with either a VTE event, death, emigration from a participating general practice or end of follow-up (Dec 31, 2010), whichever occurred earliest.

We calculated the rate of VTE according to number of VTEs per 1000 person-years of follow-up at risk. Through Cox regression we estimated Hazard ratios for VTE in cancer patients compared to controls, adjusting for sex, age at cancer diagnosis, and calendar year. All analyses were carried out using STATA version 11.2 (Statacorp, 4905 Lakeway Drive, College Station, Texas 77845, USA). P-values < 0.05 were considered statistically significant.

### Ethics

This study was approved by the CPRD Independent Scientific Advisory Committee (Protocol no. 10–091).

## Results

Four hundred and ninety eight (498) children with cancer fulfilled our case criteria and were selected to the study group. The control group comprised 20 810 children. The median age at first cancer diagnosis was 7 years, with controls being 8 years at pseudo-diagnosis, whilst 55% of cases (and 51% of controls) were male. Additional data on participant characteristics, including total and median follow-up, are given in Table 1. Of the 498 individuals with a diagnosis of cancer during childhood, some 143 (28.7%) had leukemia, 80 (16.1%) tumours of the brain and central nervous system, and 68 (13.7%) a lymphoma (Table 2).

### Risk of VTE

During follow-up, four cancer patients and four controls were diagnosed with VTE. The cancer patients had diagnoses of hematological, bone and non-specific cancers. The overall absolute VTE rate in children with cancer was 1.52 per 1000 person-years (95%CI = 0.57-4.06) compared with 0.06 per 1000 person-years (95%CI = 0.02-0.15) in controls (Table 2). Adjusting for sex and age at cancer diagnosis, this corresponded to a HR of 28.3 for VTE (95%CI = 7.0-114.5). Further data on VTE in children with cancer are given in Table 2.

## Discussion

This study found a highly increased relative risk of VTE in children with cancer compared to children without cancer. However absolute risks of thromboembolism were very small and only about 1–2 per 1000 children with cancer per year had a VTE. Given these findings it is not unreasonable for physicians to consider using thromboprophylaxis in children with cancer. However this must be tempered with the knowledge that such a strategy would, almost inevitably, invoke adverse events secondary to the drugs prescribed [18]. Notably we found that the incidence of VTE in children in general is significantly lower than in adults and as routine thromboprophylaxis is generally not used for children without other known risk factors before puberty in connection with, for example, orthopedic surgery caution must be exercised prior to acting on our findings in the clinical sphere.

### Prior Literature

Several earlier studies have reported excess risks of thromboembolism in pediatric cancer patients [6,7,19–31], but none of these studies examined the relative risk of VTE in children with cancer compared to that in a general population of children.

A single center study registry from Belarus reported that 2.1% (44/2061) children with various malignancies had VTE [6] while a Canadian study reported a higher prevalence of 7.6% (55/726) [7]. Children with hematological malignancies are exposed to asparaginase and steroid induced coagulation defects. Incidence of VTE in children with ALL varies between 1 and 36% [32,33]. An American study reported a prevalence of 5% (27/501) for VTE in children with ALL [30], consistent with a meta-analysis comprising 1752 children with ALL and an overall risk of symptomatic thrombosis of 5.2% [34]. In a randomized controlled trial where study participants underwent three ultrasound investigations in six months, some 36% of children with ALL had asyptomatic VTE [35]. Furthermore, in another study 12% (9/75) of children with lymphoma developed thrombosis [36]. Less data is available on children with other solid tumors; however, 14.3% (10/70) of children with sarcomas developed thrombosis in a retrospective Canadian study [37]. In contrast, the incidence of VTE in children with brain tumors is relatively low [38].

### Strengths and Limitations

The main strengths of this study are its population-based design, the large number of childhood cancers and the general population comparator. The cancers we studied are typical of those seen in children in the UK, and we believe therefore that our results should be generalizable. Based on almost 500 cancers in children, we had sufficient statistical power to detect an increase in risk of the magnitude we observed, but were unable to explore the VTE risk in individual cancers. Also, based on the upper confidence limit for the absolute rate our study has sufficient power to suggest that the absolute VTE rate in children is unlikely to be any higher than 0.4% per annum. Previous validation of the three registers in this study show that they are suitable for research, where the purpose is to calculate absolute and relative risks. However, these registers do not contain detailed data on all relevant confounders, and they do not contain data on mechanism of disease.

Unfortunately we also did not have any data on thromboprophylaxis such as pharmacological anticoagulation or vena cava filters [2]. However in the United Kingdom during the period of this study routine thromboprophylaxis was not in use. We did not have access to data on specific chemotherapeutic agents.

The use of ICD codes to identify VTE has recently been questioned [39]. In this study we therefore requested additional proof of VTE for a positive outcome and we believe that the risk of VTE misclassification is low. Yet, our stringent criteria may be part of the explanation for the lower VTE rates (0.06/1000 person-years) we found than reported elsewhere [40,41]. The higher rates in other studies may partly be due to surveillance bias, where patients in smaller studies are intensively monitored for VTE. Meanwhile, the VTE rate in our control population was similar to that of the general population in other countries [42]. Extending the observation period to include the months immediately before diagnosis would not have substantially changed the observed rates, as no VTE events occurred within this period.

### Potential Mechanisms

Children with cancer have several risk factors for VTE including central venous catheters (CVCs), chemotherapy, surgery infections and immobility. There are probably other mechanisms, which may be cancer specific and so explain why cancer site is important to the risk of VTE in children [7]. The excess risk of VTE in ALL is largely thought to be due L-asparaginase-induced antithrombin deficiency. In particular chemotherapy with concomitant treatment with steroids and asparaginase has been associated increased risk for thrombosis [21,34]. Most of the VTEs in ALL occur during the induction or re-induction phases.

### Clinical Implications

Given the increase in VTE in children with cancer we have observed it might seem natural to use thromboprophylaxis in this high-risk patient group [2] but benefits must be weighed against potential harm. Currently there is no consensus nor evidence that anticoagulation in children with cancer will prevent all VTE [35]. Cancer patients receiving anticoagulant treatment following a VTE are at increased risk of bleeding [43] (although some studies show only a small cumulative risk of major bleeding [44]). The studies on VTE prophylaxis in children with cancer are limited [35,45–48] and show contradictory results. To date there are no data upon which to base general recommendations for prophylactic anticoagulation for children with malignancies.

Mitchell et al. [49]. suggested a predictive scoring system for identifying children with leukemia at increased risk for thromboses. Their method incorporates three leukemia protocols with different induction phases, CVCs and inherited thrombophilia. Of the 339 children with ALL, they identified 19 with high VTE risk. Of those, 8 received prophylactic LMWH during induction and had a significantly lower thrombosis rate than those with high VTE risk without LMWH. However, as inherited prothrombotic defects are rare and practically all children with cancer have CVCs, the group with high VTE risk with this approach will be limited.

Our study confirms the high thrombosis risk in children with sarcomas observed previously in the Canadian study [37], although our estimate has wide confidence intervals. While brain tumours constitute the second most frequent type of cancer in children [1], none of the 80 patients with brain or CNS tumours in our study had a VTE during follow-up. The lack of VTE in children with brain tumours is consistent with the lower risk of VTE in children with brain tumours observed previously [7]. A recent review [2] found only four smaller studies on VTE treatment in brain tumour patients with no definitive results and hence it is beyond this study to suggest recommendations for thromboprophylaxis in individual cancers.

## Conclusion

In conclusion, children with cancer seem to be at a highly increased risk of VTE, but absolute risks were small in the current study with less than 1% of children having a VTE during an average follow-up of 5 years. Considering earlier reports of an association between thromboprophylaxis and bleeds, more information is needed before instituting general thromboprophylaxis in children with cancer.

## Role of the Funding Source

This study was funded by a project grant from Cancer Research UK (Ref: C17683/A12079)

JW was funded by a Nottingham University/Nottingham University Hospitals NHS Trust Senior Clinical Research Fellowship.

JFL was supported by grants from the Swedish Society of Medicine and the Swedish Research Council (SIMSAM).

*None of the funders had any influence on this study.*

## Author Contributions

Study concept and design: AJW, MJG, TRC, JW, SR, JFL

Acquisition of data: AJW and MJG

Analysis and interpretation of data: AJW, MJG, TRC, JW, SR, JFL

Wrote the first draft of the manuscript: JFL

Critical revision of the manuscript for important intellectual content: AJW, MJG, TRC, JW, SR, JFL

Statistical analysis: AJW

Study supervision: JFL and MJG

## Conflict of Interest

No conflicts of interest to declare.

## Figures and Tables

**Table 1:** Characteristics of study participants.

		Cancer patients	% (IQR)	Controls	% (IQR)
Total		498		20 810	
Median age (years)		7	(3–13)	8	(3–12)
Sex	Male	273	54.8	10 694	51.4
	Female	225	45.2	10 116	48.6
Follow up time (years)	Total	2 627		68 761	
	Median	5.0	(2.2-8.0)	2.1	(0.8-5.0)
VTE⁎	No	494	99.20	20 806	99.98
	Yes	4	0.80	4	0.02

IQR, Interquartile range.

⁎ VTE, Venous thromboembolism.

**Table 2:** Risk of venous thromboembolism in children with cancer.

	No of children	Rate per 1000 person-years; 95% CI
Controls (no cancer)	20 810	0.06; 0.02-0.15
All cancers	498	1.5; 0.6-4.1
Leukemias/Lymphomas	211	0.9; 0.1-6.1
Brain & CNS⁎	80	0
Soft tissue sarcoma/Bone	54	8.1; 2.0-33.0
SNS tumours#	8	0
Renal	43	0
Carcinoma & malignant melanoma	18	0
Gonadal & germ cell	15	0
Retinoblastoma	7	0
Hepatic	8	0
Unknown	3	0
Other site	52	4.0; 0.6-29.0

⁎ CNS, Central nervous system. # SNS, Sympathetic nervous system.
